# P02.88. Powdered Red Yeast Rice and Plant Stanols and Sterols to lower cholesterol

**DOI:** 10.1186/1472-6882-12-S1-P144

**Published:** 2012-06-12

**Authors:** J Feuerstein, W Bjerke

**Affiliations:** 1 Stamford Hospital, Columbia University, Stamford, USA; 2 Saced Heart University, Fairfield, USA

## Purpose

Elevated Low-Density Lipoprotein (LDL) cholesterol is a significant risk factor for cardiovascular disease. It is estimated that 42% of females and 34% of males in the USA have elevated total cholesterol. The current mainstay of lipid lowering therapy utilizes hydroxy-methyl-glutaryl-coenzyme A reductase inhibitor (‘statin’) medications that lower total cholesterol and LDL cholesterol by 20% and 28%, respectively. However, due to the significant side effects of statin medications, many patients seek alternative therapies to help manage their hypercholesterolemia. Red Yeast Rice Monascus Purpueus has been used as a food and as an herbal medication in China for centuries. Plant Stanols and Sterols are foods that are similar in structure and function to animal cholesterol. Both these compounds have been shown in clinical studies to significantly lower LDL.

## Methods

We report on a case series of 18 patients with hypercholesterolemia despite therapeutic lifestyle change through diet and exercise who took a proprietary product combining Red Yeast Rice and Plant Stanols and Sterols as a powdered shake in an effort to improve their cholesterol.

## Results

Statistically significant reduction (p<0.05) in the following mean variables were seen: total cholesterol 19% (46 mg/dL) and LDL 33% (53mg/dL) after 6 weeks using the blend. There was no significant difference in BMI, triglycerides, HDL cholesterol levels, or SBP and DBP over the same period.

## Conclusion

This magnitude of reduction in LDL cholesterol is significantly greater than the 28% reduction observed in the 1999 JAMA meta-analysis on the effectiveness of statin medications in lowering cholesterol levels. None of the participants in our study reported any muscle pains and no abnormal liver function tests were seen whilst taking the blend. Though this case series is limited by small sample size and study duration, the blend’s significant reduction in LDL cholesterol without severe side effects indicates that this product is an effective alternative to statins.

